# Surgery of hereditary hemorrhagic telangiectasia with severe refractory gastrointestinal bleeding: A case report of a rare condition

**DOI:** 10.1016/j.ijscr.2020.06.057

**Published:** 2020-06-13

**Authors:** Dae Ro Lim, Da Bin Kim, Hee Kyung Kim, Eung Jin Shin

**Affiliations:** a Division of Colon and Rectal Surgery, Department of Surgery, Soonchunhyang University Bucheon Hospital, Bucheon, South Korea; b Department of Pathology, Soonchunhyang University College of Medicine, Soonchunhyang University Bucheon Hospital, Bucheon, South Korea

**Keywords:** Hereditary hemorrhagic telangiectasia, Gastrointestinal bleeding, Surgery

## Abstract

**Introduction:**

Hereditary hemorrhagic telangiectasia (HHT) is an autosomal dominant genetic disease that affects the vasculature of numerous organs. HHT is characterized by the presence of multiple arteriovenous malformations that lack intervening capillaries and result in direct connections between arteries and veins.

**Presentation of case:**

A 58-year-old South Korean female was admitted presenting with severe anemia, dizziness, and intermittent hematochezia. The patient had experienced chronic severe refractory gastrointestinal bleeding for years. She had an intermittent spontaneous epistaxis, telangiectasias in the oral cavity (tongue), and hepatic arteriovenous malformations and gastrointestinal telangiectasia. She underwent a segmental resection of a segment of the ileum and cecum (about 77 cm). After surgery, the patient experienced no more drops in hemoglobin count or symptoms of anemia and melena. She is living a normal life.

**Conclusion:**

The present case report is a surgical case of undiagnosed HHT with chronic refractory gastrointestinal bleeding. Surgical treatment is rarely required for gastrointestinal bleeding unless nonsurgical methods such as endoscopy/colonoscopy and angiography have failed.

## Highlights

• Hereditary hemorrhagic telangiectasia (HHT) affects the vascular structure of numerous organs.
• Clinical symptoms of HHT is epistaxis, GI bleeding, and iron deficiency anemia caused by mucocutaneous telangiectasias.
• The treatment of HHT with gastrointestinal bleeding may need surgical treatment.

## 1 Introduction

Hereditary hemorrhagic telangiectasia (HHT), also called Osler-Weber-Rendu syndrome, is an autosomal dominant genetic disease that affects the vascular structure of numerous organs. HHT is characterized by the presence of several arteriovenous malformations, which lack mediated capillaries and form direct connections between arteries and veins [1]. The prevalence of HHT was estimated at 1:5,000–1:8,000, roughly the same as in European and US populations, and there is a traditional view among the Asian medical profession that HHT is rare [1]. The prevalence of HHT in the UK is approximately 1 in 9400 [2], and the overall prevalence of HHT in North America is estimated to be 1:10,000 [3]. There is still a lot to learn about this disease, but the understanding of its pathophysiology, genetic basis, presentations, and management is increasing. Patients typically present clinical symptoms of epistaxis, gastrointestinal bleeding, and iron deficiency anemia caused by mucocutaneous telangiectasias [4]. In addition to clinical symptoms, HHT can also result in gastrointestinal telangiectasias, as well as large arteriovenous malformations of the lung, brain, and liver [4]. The present case report is a surgical case of HHT with chronic refractory gastrointestinal bleeding that was not previously diagnosed as HHT. This work has been reported in line with the SCARE criteria [5].

## 2 Case report

**Fig. 1:**
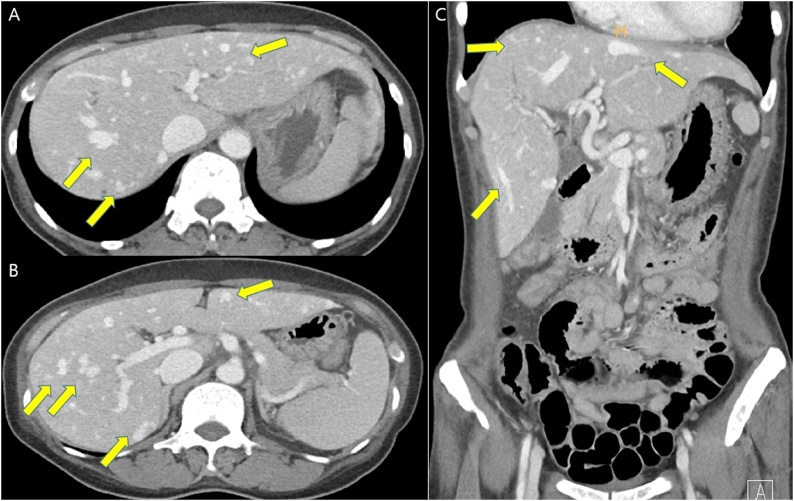
Abdominopelvic CT scan: A & B) Dilated tortouous hepatic arteries with mutifocal arterio-venous shunts in liver, arterio-venous malformation in ileal wall. C) Coronal view.

**Fig. 2:**
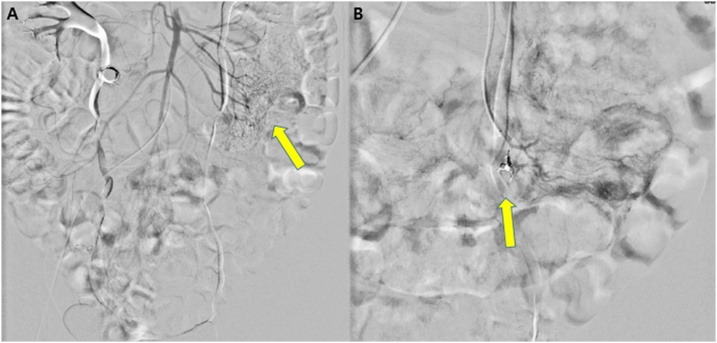
Angiography: A) Contrast extravasation from proximal ileum. B) Transcatheter arterial embolization was two times performed for selective embolization of two ileal branch using 1 via gelfoam particle and 1 microcoli, respectively.

A 58-year-old South Korean female was admitted presenting with anemia, dizziness, and intermittent hematochezia. She had a history of iron deficiency anemia beginning about 5 years previously. She had been admitted to hospital three times over the past 4 years because of anemia (hemoglobin count 5–7 g/dL) with intermittent nasal bleeding (epistaxis) and melena or hematochezia. She had sometimes undergone transfusion with packed red blood cells (RBCs). She had undergone a clipping of multiple angiodysplasias in the antrum and body of the stomach by gastroenteroscopy 4 years previously. At the time of most recent admission, the laboratory testing revealed a white blood cell count of 4850 cells/mm^3^ and a hemoglobin count of 4.8 g/dL. The other routine laboratory tests revealed no specific findings. Her clinical symptoms were dizziness, general weakness, and intermittent hematochezia. A gastroenteroscopy and colonoscopy were performed and revealed no specific concerns. Abdominopelvic CT scan showed dilated tortuous hepatic arteries with multifocal arteriovenous shunts in the liver and arteriovenous malformation in the ileal wall. A gastrointestinal bleeding scan revealed active bleeding in the left- to mid-pelvic cavity (ileum) (Fig. 1). Angiography was performed and revealed contrast extravasation from the proximal ileum. Subsequently, transcatheter arterial embolization was twice performed for selective embolization of two ileal branches, using Gelfoam® (Pfizer, Kalamazoo, Michigan, US) particles and microcoil, respectively (Fig. 2).

**Fig. 3:**
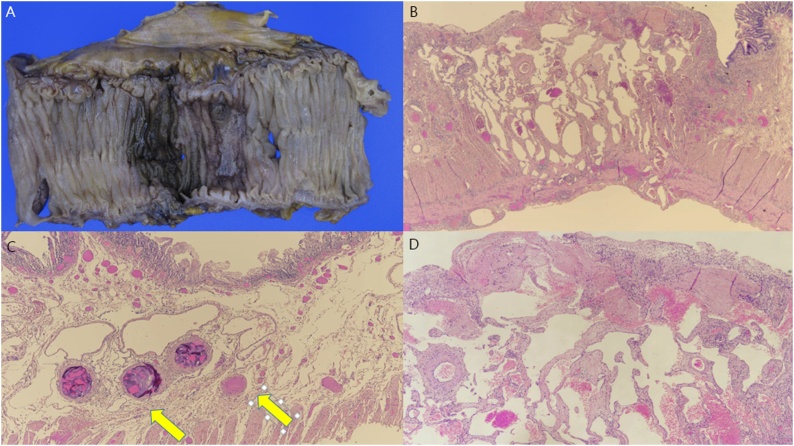
Macroscopically, mucosal denudation, submucosal congestion and diffuse wall thinning, consistent with ischemic change was found (A). Histologically, focal abnormal ectatic vascular proliferation in the entire intestinal wall, centered at the submucosa, suggestive of angiodysplasia was found (B, C-arrows, D).

However, the patient’s symptoms did not improve. She continued to show low levels of hemoglobin and intermittent even after bleeding control by transcatheter arterial embolization using angiography. She underwent a segmental resection of the segment of the ileum and cecum (about 77 cm) that had been marked (embolized material) previously during angiography. The intraoperative investigation yielded no remarkable findings except for the embolized material of the ileal branch and no palpable masses. Macroscopically, mucosal denudation, submucosal congestion, and diffuse wall thinning consistent with ischemic change were found (Fig. 3A). Histologically, focal abnormal ectatic vascular proliferation in the entire intestinal wall, centered at the submucosa, suggestive of angiodysplasia was found (Fig. 3B–D). After surgery, the patient experienced no more drops in hemoglobin count (10.4 g/dL) or symptoms of anemia and melena. At the last follow-up visit (after 3 months), her hemoglobin count was normal (10.0 g/dL) and she was living a normal life. She then visited the outpatient clinic 6 months after surgery. Her hemoglobin count was 10.1 g/dL and she had no symptoms.

## 3 Discussion

**Fig. 4:**
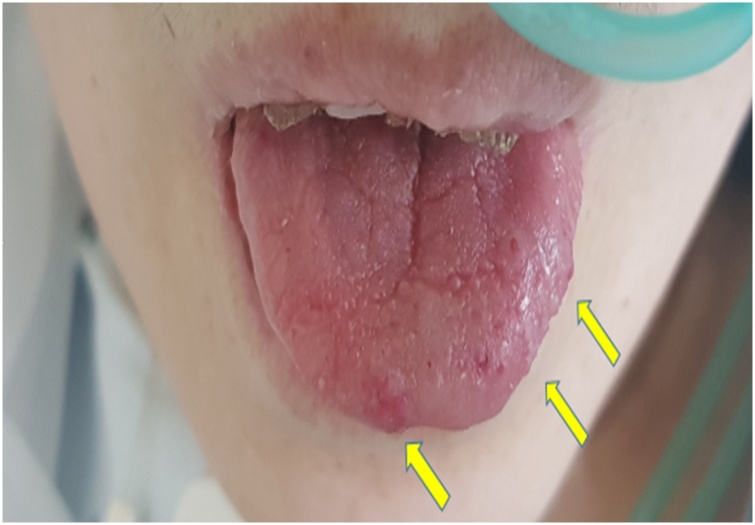
Telangiectasias was found in oral cavity (tongue) in present patients with HHT.

HHT is a familial disease that causes systemic abnormalities in the capillary veins. Two major mutations of chromosome 9 (HHT1) and 12 (HHT2) have been identified respectively in the genes encoding the endoglin and the activin receptors such as kinase [6]. HHT is a rare disease and its diagnosis is rather difficult. It is usually diagnosed clinically; a definitive diagnosis of HHT can be made when three or more criteria are satisfied under the Curaçao criteria and should be suspected when two criteria are satisfied [7]. The Curaçao criteria (international consensus diagnostic criteria) are the mainstay of diagnosis of HHT in adults. The criteria include spontaneous and recurrent epistaxis; telangiectases at characteristic sites (oral cavity, nose, and fingers); visceral involvement (gastrointestinal telangiectasia, pulmonary arteriovenous malformations, hepatic arteriovenous malformations, cerebral arteriovenous malformations); and a first-degree relative with HHT [7,8]. Patients with HHT present with various symptoms and various involvements of telangiectasia that gradually develop with age. By the age of 16, about 70% of individuals have some features of HHT, and by the age of 40, this figure becomes 90% [9]. The present case satisfied three Curaçao criteria. The patient had 1) an intermittent spontaneous epistaxis, 2) Telangiectasias in the oral cavity (tongue) (Fig. 4), and 3) hepatic arteriovenous malformations and gastrointestinal telangiectasia. The patient had a history of intermittent spontaneous epistaxis and iron deficiency anemia with melena lasting several years. The patient’s tongue currently has multiple maroon-colored telangiectasias. Finally, the patient’s CT scan showed dilated tortuous hepatic arteries with multifocal arteriovenous shunts in the liver and arteriovenous malformation in the ileal wall. Epistaxis and gastrointestinal bleeding are the most common causes of chronic anemia in patients with HHT. Gastrointestinal bleeding is present in about 13–30% of cases and it usually presents in the patient’s fifties [10]. The present case’s age also falls within the fifth decade and recorded gastrointestinal bleeding.

There is no clear definitive treatment for patients with HHT’s gastrointestinal bleeding. The treatment options depend on the severity of symptoms and the site of gastrointestinal bleeding. If active bleeding is present and the bleeding site is in the upper or lower gastrointestinal tract (esophagus, stomach, duodenum, colon), endoscopic/colonoscopic therapy could be applied. One case report described a case of bleeding control using endoscopic argon beam coagulation [11]. However, in cases for whom endoscopic therapy fails or is impossible and there is no response to angiography, surgery may be necessary, as in the present case. The present case’s bleeding site was the ileal mesentery, which could not be controlled by endoscopic/colonoscopic therapy. In addition, bleeding was not controlled by angiography. In chronic refractory gastrointestinal bleeding, medical treatment may be effective. Thalidomide and bevacizumab are antiangiogenic drugs that have been shown to improve recurrent nose bleedings and incidences of telangiectasia. Bevacizumab affects the vascular endothelial growth factor receptors and prevents abnormal angiogenesis. Long-term treatment with intravenous bevacizumab has shown an improvement in the cessation of bleeding and improved the hemoglobulin level [12,13]. Thalidomide is an antiangiogenic and immunomodulatory agent; it may modulate the activation of mural cells and embrace blood vessels, effectively reduce epistaxis and transfusion requirements, and ameliorate anemia in HHT patients [14,15].

In conclusion, the present case report is a surgical case of undiagnosed HHT with chronic refractory gastrointestinal bleeding. The treatment of HHT with gastrointestinal bleeding may include medical therapy or nonsurgical methods such as endoscopy/colonoscopy and angiography, but there are cases where surgical treatment is required.

## Declaration of Competing Interest

The authors declare that they have no conflicts of interest with respect to this work.

## Funding

This work was supported by the 10.13039/501100002560Soonchunhyang University Research Fund.

## Ethical approval

This manuscript is a case report retrospectively and also is not a clinical study. The ethical approval is not necessary.

## Consent

The patients have provided written informed consent for publication of the case.

## Author contribution

Dae Ro Lim contributed writing the paper, study concept, data collection.

Da Bin Kim contributed data collection and data analysis.

Hee Kyung Kim contributed data analysis and interpretation.

Eung Jin Shin contributed study concept or design.

## Registration of research studies

This manuscript is a case report retrospectively and also is not a clinical study.

## Guarantor

This work was supported by the Soonchunhyang University Research Fund.

## Provenance and peer review

Not commissioned, externally peer-reviewed.
